# The antimicrobial peptide KR-12 promotes the osteogenic differentiation of human bone marrow stem cells by stimulating BMP/SMAD signaling

**DOI:** 10.1039/c8ra00750k

**Published:** 2018-04-24

**Authors:** Hui Li, Shutao Zhang, Bin'en Nie, Zhe Du, Teng Long, Bing Yue

**Affiliations:** Department of Bone and Joint Surgery, Renji Hospital, Shanghai Jiaotong University School of Medicine 145, Shandong Road Shanghai 200011 China advbmp2@163.com +86-21-53882199

## Abstract

KR-12 is the smallest fragment of human antimicrobial peptide cathelicidin (LL-37), and could play key roles in the treatment of multiple infections, including osteomyelitis. Our preliminary work found that KR-12 enhances the osteogenic differentiation of human bone marrow mesenchymal stem cells (HBMSCs). The present study investigated whether KR-12 affects HBMSC osteogenic differentiation, as well as the molecular mechanisms involved. HBMSC proliferation in the presence of KR-12 was observed with a cell counting 8 assay, and its effects on HBMSC cell cycle progression and apoptosis were examined by flow cytometry. Alkaline phosphatase, Sirius Red, and Alizarin Red staining and quantitative assays were used to study the osteogenic differentiation of HBMSCs. The expression of osteogenic differentiation markers was detected by real-time quantitative PCR analysis. The activation of potentially related pathways was examined by luciferase reporter assay and western blot analysis. KR-12 treatment increased the osteogenic differentiation of HBMSCs without cytotoxicity and did not influence the cell cycle or induce apoptosis. Luciferase reporter assays showed that KR-12 activated the transcription of bone morphogenetic protein 2 (BMP2), a key gene in the BMP/SMAD pathway. Western blot analysis indicated that BMP/SMAD signaling was markedly activated by KR-12 stimulation in osteogenic induction conditions. SMAD phosphorylation was activated by KR-12 treatment, and was inhibited by both a transforming growth factor-β/SMAD inhibitor (LDN-193189 HCL) and BMP2 small interfering RNA (si-BMP2). LDN-193189 HCL and si-BMP2 treatment also abolished the KR-12-induced osteogenic differentiation of HBMSCs. In conclusion, our results suggest that KR-12 promotes HBMSC osteogenesis through the activation of BMP/SMAD signaling.

## Abstract 2

KR-12 is the smallest fragment of human antimicrobial peptide cathelicidin (LL-37), and could play key roles in the treatment of multiple infections, including osteomyelitis.

## Graphical abstract

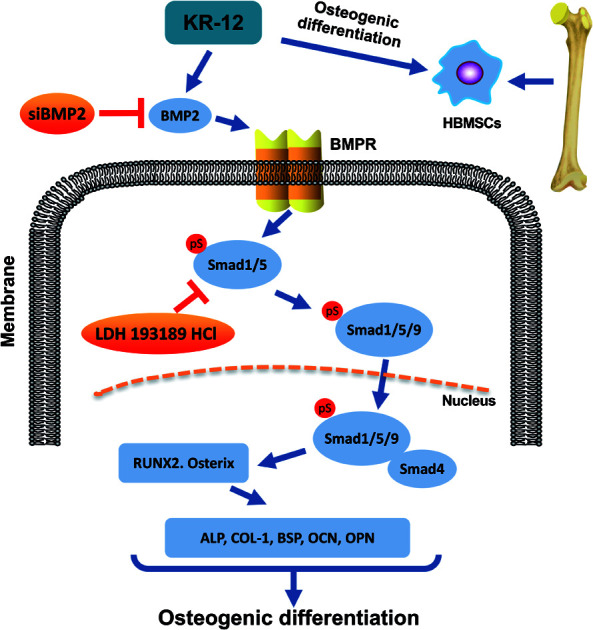

## Introduction

Antimicrobial peptides (AMPs) are components of the innate immune system and have been identified in almost all living organisms, from plants to insects to mammals, including humans. Because of their ability to physically disrupt microbial membranes and induce lysis, AMPs can target bacteria, fungi, and enveloped viruses and are potential alternatives to conventional antibiotics.^1,2^ The only natural antibacterial peptide in the human body, cathelicidin (hCAP18/LL-37), was identified in 1995;^3–5^ this antimicrobial protein has been detected in various cell types and epithelial surfaces,^6,7^ including the gut epithelia,^8,9^ mast cells,^10^ monocyte subpopulations, and lymphocytes.^11^ Human cathelicidin consists of a conserved N-terminal pro-sequence called the cathelin-like domain (hCAP18) and a highly variable C-terminal antimicrobial region called LL-37 (LLGDFFRKSKEKIGKEFKRIVQRIKDFLRNLVPRTES), and is an important first line of defense against local infection, systemic pathogen invasion, and wound inflammation.^6,7,12,13^ However, LL-37 is too long to develop as a therapeutic agent for bacterial infections and inflammatory diseases.^14^ Short AMPs attract attention because of their lower production cost. In addition, removing hydrophobic amino acids at the N-terminus of native LL-37 reduces its toxicity to eukaryotic cells and decreases its interaction with human plasma proteins.^15^ Two LL-37 derivatives of 20 and 30 amino acids, named KR-20 (KRIVQRIKDFLRNLVPRTES; 18–37) KS-30 (KSKEKIGKEFKRIVQRIKDFLRNLVPRTES; 8–37) have been found in human sweat. These three peptides exhibit increased antimicrobial activity compared with the native LL-37.^16^ Additionally, LL-37 fragments of different lengths have been tested for activity against bacteria, fungi, and cancer cells. KR-12 (KRIVQRIKDFLR; 18–29) is the shortest peptide that demonstrates antibacterial activity.^17–19^ This peptide retains the core amphipathic helix structure of LL-37 and contains 5 cationic residues.^20^ Compared with LL-37 and its other derivatives, KR-12 is less costly to synthesize and has lower cytotoxicity. LL-37 has a certain degree of hemolytic activity against human red blood cells, but KR-12 does not induce red blood cell hemolysis.^14,20^ Therefore, KR-12 could be a promising candidate for preventing and treating both local and systemic infections.

Osteomyelitis is a common infection that causes symptoms of varying severity, either locally or systemically. Bone infections can have serious consequences, like protracted infected lesions, necrotic bone formation, implant loosening, and surgical failure, and often require great treatment efforts.^21^ In addition to surgical removal of the infected bone, local or systemic antibiotics are the most common treatments.^22,23^ Antibiotics commonly used in the treatment of bone-related infections include β-lactams, cephalosporins, aminoglycosides, macrolides, and quinolones, resulting in increased emergence of bacterial resistance.^24^ In addition, despite gradual control of the infection and absorption of necrotic bone tissue, osteolysis resulting from the infection cannot be resolved by traditional antibiotics. A commonly used antibiotic for bone-related infections, gentamicin has been shown to inhibit osteoblast viability and cell number.^25^ Analogously, vancomycin inhibits osteoblast proliferation at certain concentrations.^26^ Recently, it was reported that some small molecule peptides promote osseointegration while controlling infection.^27^ The discovery of agents with good antibacterial properties that can also promote osseointegration would be clinically significant.

Human bone marrow mesenchymal stem cells (HBMSCs) play a key role in bone renewal. They can differentiate into bone-forming osteoblasts, and are a prime source of osteoprogenitor cells.^28–30^ They can also be used in grafting materials to treat bone defects.^31^ HBMSCs can be induced to differentiate into osteoblasts by the addition of dexamethasone (DEX), ascorbic acid, and β-glycerophosphate,^32,33^ and the stimulation of osteogenic differentiation and osteoblast function increases bone formation. After a local infection is cleared, HBMSCs are activated and differentiate into osteoblasts to repair local bone dissolution. If the HBMSCs fail to completely repair local bone defects caused by infection, it can cause local osteoporosis and pathologic fracture.^34^ Currently, drugs that function as bone absorption inhibitors by suppressing osteoclast activity such as vitamin D analogues, calcitonin,^35^ and estrogen,^36^ are used in osteoporosis treatment.^37^ However, these drugs do not promote osteogenic differentiation, nor do they possess antibacterial properties.

Bone morphogenetic proteins (BMPs) are members of the transforming growth factor (TGF-β) superfamily. They are potent osteoblastic differentiation factors during bone formation, playing a significant regulatory role.^38,39^ Intracellular BMP signal transduction is mediated mainly by SMAD proteins, which are activated by BMP binding to transmembrane receptor serine–threonine kinases. Activated SMADs translocate to the nucleus, where they modulate the expression of osteoblast-related genes.^40^ BMP2 can induce bone formation and differentiation *in vivo* and *in vitro*.^41^ It activates SMAD1/5/8 and several mitogen-activated protein kinases (MAPKs) including extracellular regulated kinase (ERK)1/2, p38, and c-Jun kinases,^42–45^ ultimately resulting in increased expression of runt-related transcription factor 2 (RUNX2) and core binding factor (CBFA1).^46–48^

Given the worrisome increases in clinical antibiotic resistance and the promising antibacterial properties of AMPs, we hypothesized that they may have positive effects on osteomyelitis infections. As a small antibacterial peptide, with several advantages in synthesis and biological properties, KR-12 is a good prospect for the treatment of infectious symptoms caused by osteomyelitis. What's more, in our previous study on covalent bonding of KR-12 onto titanium surface, it was found that titanium bonding with KR-12 could promote HBMSCs' osteogenic differentiation.^49^ Some articles also reported that antimicrobial peptides may have the function of promoting differentiation of stem cells.^50–53^ So we wonder whether KR-12 has the effect of promoting osteogenic differentiation of HBMSCs. In this study, we have investigated the effects of KR-12 on the osteogenic differentiation of HBMSCs and examined the molecular mechanisms involved. Given the importance of BMP/SMAD signaling in osteoblast differentiation and bone formation, we hypothesized that this pathway was involved in KR-12-induced HBMSCs osteogenic differentiation. To investigate this, we examined the effects of different concentrations of KR-12 on both HBMSC osteogenic differentiation and BMP/SMAD signaling.

## Materials and methods

### 1. Reagents

KR-12 (KRIVQRIKDFLR) was synthesized and purified by GL Biochemistry (Shanghai). Alizarin Red dye solution was purchased from Servicebio (Wuhan, China). Phosphatase (ALP) Color Development Kit, ALP Quantitative Kit, RIPA lysis buffer, and phenylmethylsulfonyl fluoride (PMSF; 100×) were purchased from Beyotime Biotechnology (Nantong, China). TRIzol reagent was purchased from Invitrogen (Carlsbad, CA, USA). The SYBR Premix EX Taq Real-time PCR kit and cDNA Synthesis Kit were purchased from TaKaRa (Japan). Protease inhibitor cocktail (100×) and the BCA Protein Assay Kit were purchased from Thermo Fisher (USA). Polyvinylidene fluoride membrane for western blotting and Immobilon western Chemiluminescent HRP Substrate (ECL) were purchased from Millipore (USA). Monoclonal antibodies against SMAD1 (D59D7), SMAD5 (D4G2), SMAD4 (D3M6U), P-SMAD1/5 (41D10), GAPDH (D16H11), and horseradish peroxidase-linked anti-IgG secondary antibodies were purchased from Cell Signaling Technology (USA). TGF-β/SMAD inhibitor (LDN-193189 HCL) was purchased from Selleck Chemicals (Houston, TX, USA). Lipofectamine™ 3000 and Lipofectamine™ RNAiMAX transfection reagent were purchased from Invitrogen (Carlsbad, CA, USA). *Gaussia* luciferase reporter plasmids and Secrete-Pair Assay Kit were purchased from Genecopoeia (Rockville, MD, USA).

### 2. Cell culture

HBMSCs were isolated and expanded as previously described.^54^ All experiments were performed in accordance with the Guidelines “Ethical review methods for biomedical research involving people”, and approved by the ethics committee at Shanghai Renji Hospital, Shanghai Jiaotong University School of Medicine. Informed consent was obtained from the human participant of this study. Seven-milliliter bone marrow aspirates were harvested in heparin (about 200 U mL^−1^ total) from the iliac crest of a healthy adult donor. Then the bone marrow was diluted in α-MEM containing 10% (v/v) FBS. The suspension was seeded into T-25 cell culture flasks at 3.5 mL of bone marrow per flask. The cultures were maintained in an incubator at 37 °C in a humidified atmosphere of 5% CO_2_, and the medium was replaced on the fourth day. The colony forming units were cultured with complete medium (α-MEM containing 10% FBS) until the cells were confluent. After digestion with trypsin-EDTA, the first generation cells were split into three T-25 cell culture flasks. The culture medium was changed every 2 days. Cells from passage 3 were used for experiments.

**Fig. 1:**
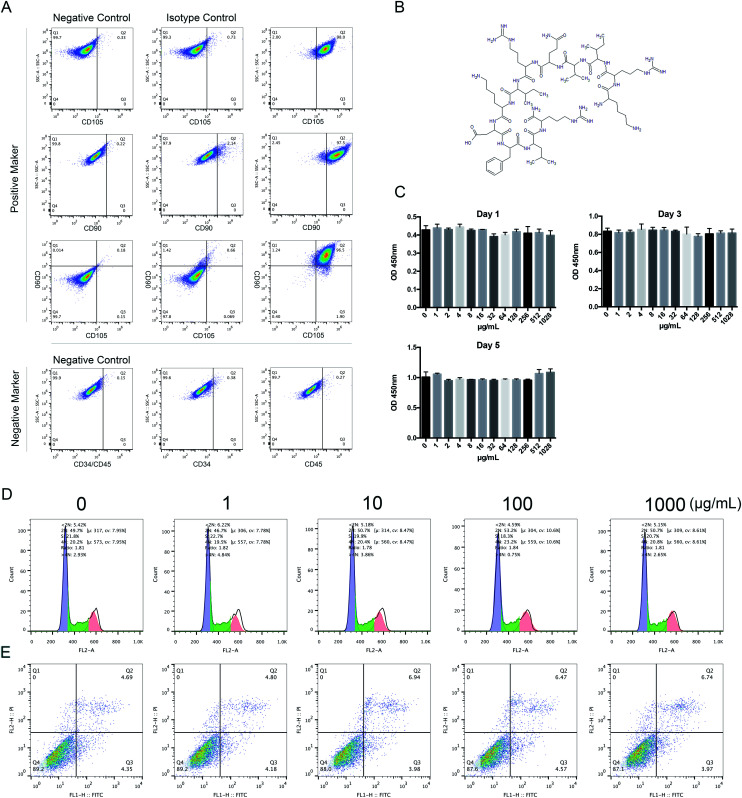
The effects of KR-12 on viability of HBMSCs. Cells were incubated with the indicated concentrations of KR-12 (0–1000 μg mL^−1^). (A) Surface marker analysis of HBMSCs by flow cytometry. The HBMSCs were positive for CD90 and CD105, while negative for CD45 and CD34. (B) Chemical structure of the KR-12 peptide. (C) HBMSCs were treated with different concentrations of KR-12 for 1 day, 3 days and 5 days prior to measuring the cell viability by a CCK-8 test (*P < 0.05). (D and E) Cell cycle and cell apoptosis was carried out by flow cytometry after 48 h incubation with different concentrations of KR-12.

To identify HBMSC phenotypes, 1 × 10^6^ adherent HBMSCs were washed in PBS and then incubated with the mouse anti-human monoclonal antibodies against CD90, CD105, CD34, and CD45. Isotype controls were used to detect nonspecific staining. Cells were incubated for 45 minutes at room temperature. The cell suspensions were then washed 3 times with PBS and analyzed using a flow cytometer (Fig. 1A).

### 3. Cytotoxicity, cell cycle, and apoptosis analysis

The cytotoxicity of KR-12 was assessed by CCK-8 assay according to the manufacturer's instructions. In brief, HBMSCs (5.0 × 10^3^ cells per well) were plated in 96-well plates with various concentrations of KR-12 (0, 1, 10, 100, and 1000 μg mL^−1^) for 1, 3, and 5 days. The cell culture medium was removed and 1000 μg mL^−1^ of CCK-8 solution was added to each well. After incubation for 2–3 hours, absorbencies were read at 450 nm on an enzyme-linked immunosorbent assay instrument.

The effect of KR-12 on the HBMSC cell cycle was determined by PI/RNase staining after 1, 3, and 5 days. Briefly, HBMSCs were harvested and washed with PBS. Then, 75% ice-cold ethanol was added dropwise with vortexing and the cells were fixed at −20 °C for 120 minutes in the dark. After fixation, cells were washed twice to remove the ethanol, resuspended in 0.5 mL PI/RNase staining buffer, and incubated in the dark for 15 minutes. Cycle fractions (G0, G1, S, and G2/M phases) were determined using a flow cytometer.

Assessment of apoptosis was performed by costaining for annexin V-fluorescein isothiocyanate (FITC), which binds to flipped phosphatidyl serine (PS) in the membrane, and PI, which binds to DNA. Cells (2–5 × 10^5^ cells per mL) were collected 48 hours after KR-12 treatment, washed twice with PBS, and resuspended in 200 μL 1× binding buffer. Then, 5 μL of annexin V-FITC and 10 μL of PI were added to the flow tube and incubated for 15 minutes at room temperature in the dark. Binding buffer (400 μL) was added to each tube, and stained cells were analyzed by flow cytometry within 4 hours.

### 4. Assessment of ALP activity and mineralized matrix formation

HBMSCs (5 × 10^4^ cells per well) were seeded in 24-well plates. After the cells began to adhere to the plates, osteogenic medium containing 10 mM β-glycerophosphate, 50 nM ascorbic acid, 100 nM DEX, and different concentrations of KR-12 (0, 1, 10, 100, 1000 μg mL^−1^) was added to induce differentiation.

For ALP staining, HBMSCs were washed twice with PBS after 7 days of treatment. After 30 seconds fixation, cells were stained with the ALP staining kit according to the manufacturer's instructions. To quantitatively analyze ALP activity, HBMSCs in each group were washed twice with PBS and lysed with Triton X-100 (1%) for 15 minutes. ALP activity was measured by absorbance at 405 nm, and normalized to the protein concentration, as determined by BCA assay.

HBMSCs treated with osteogenic medium and KR-12 for 21 days were stained with Alizarin Red to assess mineralized matrix deposition for bone nodule formation. Cells were washed 3 times with PBS and fixed with 75% ethanol for 1 hour, then stained with Alizarin Red dye solution at room temperature according to the manufacturer's instructions. Red staining (mineralized matrix deposition) was observed and recorded by a camera and pose microscope. For further quantitative analysis, a 10% chlorinated 16 alkyl pyridine solution of sodium phosphate (pH = 7) was added to dissolve the dye, and the absorbance was measured at 620 nm.

### 5. Real-time quantitative PCR (RT-PCR)

Total RNA was extracted from HBMSCs using TRIzol reagent. After the RNA concentrations were normalized, cDNA was synthesized using a kit. RT-PCR was performed using SYBR Green Master Mix with rhodamine X according to the manufacturer's instructions. Primers used for quantitative PCR were as follows: glyceraldehyde 3-phosphate dehydrogenase (GAPDH), 5′-GGAGCGAGATCCCTCCAAAAT-3′ (forward), and 5′-GGCTGTTGTCATACTTCTCATGG-3′ (reverse); RUNX2, 5′-TGGTTACTGTCATGGCGGGTA-3′ (forward), and 5′-TCTCAGATCGTTGAACCTTGCTA-3′ (reverse); ALP, 5′-ACCACCACGAGAGTGAACCA-3′ (forward), and 5′-CGTTGTCTGAGTACCAGTCCC-3′ (reverse); collage type 1 (COL-1), 5′-GAGGGCCAAGACGAAGACATC-3′ (forward), and 5′-CAGATCACGTCATCGCACAAC-3′ (reverse); bone sialoprotein (BSP), 5′-CACTGGAGCCAATGCAGAAGA-3′ (forward), and 5′-TGGTGGGGTTGTAGGTTCAAA-3′ (reverse); osteocalcin (OCN), 5′-CACTCCTCGCCCTATTGGC-3′ (forward), and 5′-CCCTCCTGCTTGGACACAAAG-3′ (reverse); osteopontin (OPN), 5′-CTCCATTGACTCGAACGACTC-3′ (forward), and 5′-CAGGTCTGCGAAACTTCTTAGAT-3′ (reverse); BMP2, 5′-GGTATCACGCCTTTTACTGCC-3′ (forward), and 5′-ACACCCACAACCCTCCACAA-3′ (reverse); and ostetix (OSX), 5′-ACACTGGGCAGACAGTCAG-3′ (forward), and 5′-CCCTTTACAAGCACTAATGG-3′ (reverse).

### 6. Western blot analysis

HBMSCs cultured in 6-well plates were lysed in RIPA lysis buffer containing 1× PMSF and protease inhibitor cocktail. After lysis on ice and centrifugation, supernatant protein concentrations were determined by BCA assay and normalized. Equal amounts of protein were separated on 10% sodium dodecyl sulfate-polyacrylamide gels and transferred to polyvinylidene fluoride membranes. After blocking nonspecific sites with skim milk, membranes were incubated with primary antibodies against SMAD1, SMAD5, SMAD4, P-Smad1/5, and GAPDH. After incubation with secondary antibodies in Tris-buffered saline containing Tween 20 at room temperature for 1 hour, immunoreactive bands were visualized using the ECL detection system.

### 7. Luciferase reporter genes and luciferase assay

HBMSCs were transfected using Lipofectamine 3000 following the manufacturer's instructions. The BMP2 promoter-luciferase reporter consisted of 1108 bp from the BMP2 5′ promoter region upstream of the *Gaussia* luciferase gene. As a negative control, a reporter vector without the BMP2 promoter sequence was transfected. Secreted *Gaussia* luciferase was assayed in culture media collected at days 1, 4, and 7 using the Secrete-Pair Assay Kit, according to the manufacturer's instructions. Luciferase activity was normalized to the negative control.

### 8. siRNA transfection

BMP2-specific and -nonspecific siRNA duplexes modified with Chol-OMe-Cy5 were synthesized by GenePharma Co. Ltd. (Shanghai, China). The sequences of the siRNA oligonucleotides were as follows: si-BMP2, 5′-UUCUCCGAACGUGUCACGUTT dTdT-3′; scrambled nontargeting siRNA, 5′-ACGUGACACGUUCGGAGAATT dTdT-3′. Transfection was performed according to the manufacturer's instructions.

### 9. Statistical analysis

The data are expressed as the mean ± standard deviation from at least 3 independent experiments. The results were analyzed *via* Student's *t*-test or one-way analysis of variance using the SPSS 13.0 software (SPSS Inc., Chicago, IL, USA). *P* < 0.05 indicated a significant difference between groups.

## Results

### 1. Effects of KR-12 on HBMSC proliferation, cell cycle progression, and apoptosis

The chemical structure of KR-12 is shown in Fig. 1B. To investigate the potential cytotoxic effects of KR-12, its effect on HBMSC proliferation was tested by CCK-8 assay. As shown in Fig. 1C, KR-12 was not cytotoxic at concentrations up to 1000 μg mL^−1^ after 24, 72, and 120 hours. Compared with the control group, cell cycle analysis demonstrated that different concentrations of KR-12 had no significant effect on HBMSC cell cycle progression after incubation for 48 hours (Fig. 1D). KR-12 did not induce apoptosis compared to the control group, and no significant difference was observed at any concentration after 48 hours (Fig. 1E).

### 2. KR-12 enhanced the osteogenic differentiation of HBMSCs

**Fig. 2:**
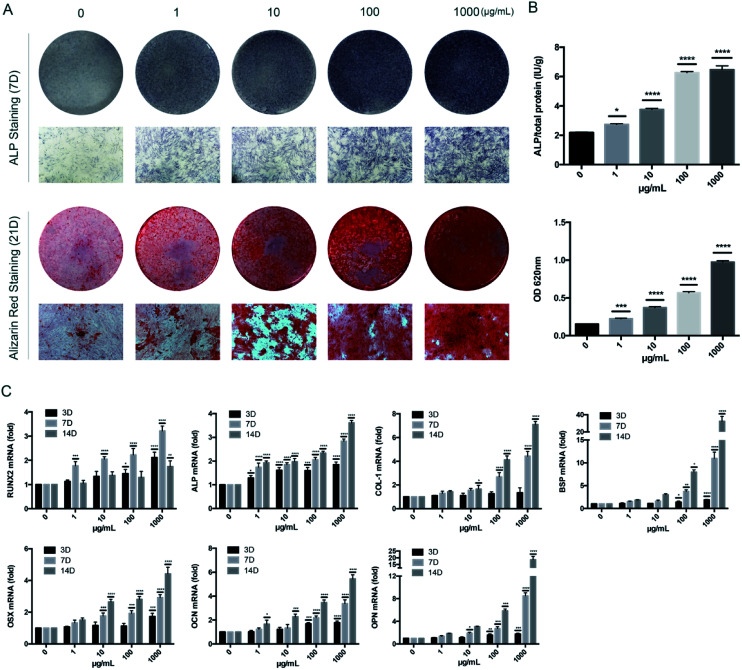
The effect of KR-12 on osteogenic differentiation of HBMSCs. Cells were incubated with osteogenic differentiation medium with the indicated concentrations of KR-12 (0–1000 μg mL^−1^). (A) Entire plate views and micrographs of alkaline phosphatase (ALP) staining at 7 days and alizarin red staining at 21 days. (B) Quantitative evaluation of ALP activity and alizarin red staining results (*P < 0.05, ***P < 0.001, ****P < 0.0001). (C) Runx2, ALP, COL-1, BSP, OSX, OCN and OPN mRNAs were subjected to real-time PCR analysis at 3, 7 and 14 days. The expression levels were normalized to that of GAPDH (*P < 0.05, **P < 0.01, ***P < 0.001, ****P < 0.0001).

To determine the influence of KR-12 on the osteogenic differentiation of HBMSCs, ALP and Alizarin Red staining and quantitative tests were performed. As the concentration of KR-12 increased, the degree of ALP and Alizarin Red staining also increased, with the highest concentration (1000 μg mL^−1^) showing the highest degree of staining (Fig. 2A). Quantitative tests showed similar results (Fig. 2B). Moreover, the mRNA expression of osteogenesis differentiation markers including RUNX2, ALP, COL-1, BSP, OPN, OCN, and OSX, were determined by RT-PCR. RUNX2 mRNA levels increased in a concentration-dependent manner after 3 days of KR-12 treatment. At day 7, ALP, COL-1 and BSP expression in the KR-12-treated groups showed obvious changes compared with the control group, the mRNA levels of OSX, OCN, and OPN started to increase. By day 14, the OSX, OCN, and OPN levels had increased significantly (Fig. 2C).

### 3. Activation of BMP/SMAD signaling in HBMSCs

**Fig. 3:**
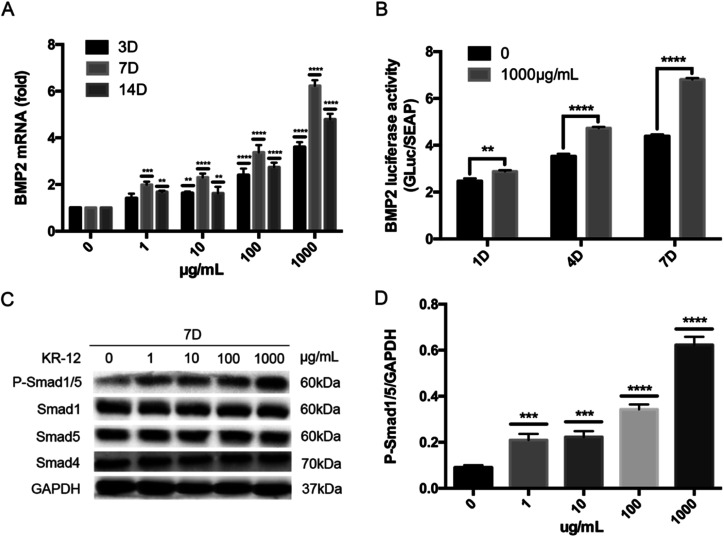
The effect of KR-12 on activation of BMP/Smad signaling during the osteogenic differentiation of HBMSCs. (A) BMP2 mRNAs levels at 3, 7 and 14 days were analyzed by quantitative real-time PCR and normalized to that of GAPDH (**P < 0.01, ***P < 0.001, ****P < 0.0001). (B) The luciferase activities of BMP2 promoter were detected at 1, 4 and 7 days, and normalized to the negative control (**P < 0.01, ****P < 0.0001). (C) The levels of phosphorylated Smad1/5, overall Smad1, Sma5 and Smad4 at 7 days were examined *via* western blotting. (D) The quantitative assay of P-Smad1/5/GAPDH (***P < 0.001, ****P < 0.0001).

We hypothesized that BMP/SMAD signaling plays an important role in KR-12-induced HBMSC osteogenic differentiation. To evaluate this hypothesis, the effects of different concentrations of KR-12 on BMP/SMAD signaling were determined *via* RT-PCR, western blot analysis, and luciferase reporter gene assays. As shown in Fig. 3A, KR-12 activated BMP2 gene expression in a dose-dependent manner. The activation of downstream components of the SMAD signaling pathway was examined by western blot analysis during HBMSC osteogenesis. KR-12 promoted the phosphorylation of SMAD1/5 in a dose-dependent manner after 7 days (Fig. 3C and D). However, the expression levels of SMAD1, SMAD4, and SMAD5 were unchanged after KR-12 treatment (Fig. 3C). We analyzed BMP2 transcription in HBMSCs treated with 1000 μg mL^−1^ KR-12 by luciferase activity assay (Fig. 3B). Compared with the control group, 1000 μg mL^−1^ KR-12 stimulated BMP2 transcription at days 1, 4, and 7. These findings suggest that KR-12 activates BMP/SMAD signaling in a concentration-dependent manner, showing marked changes at 1000 μg mL^−1^.

### 4. Inhibition of BMP/SMAD suppresses KR-12-induced osteogenic differentiation

**Fig. 4:**
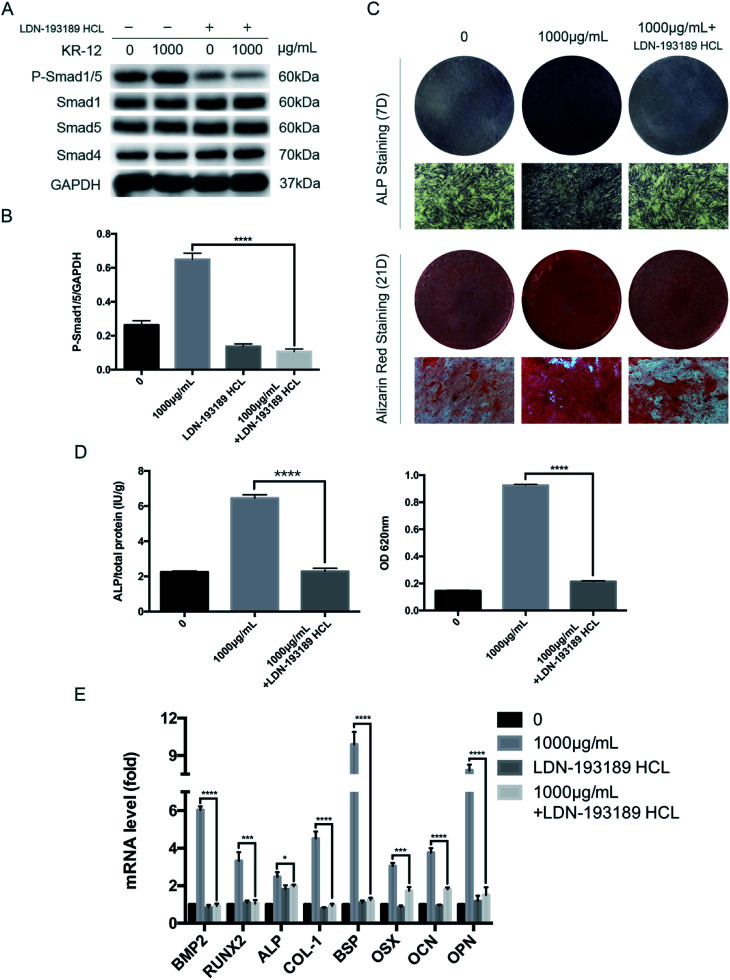
The effect of TGF-β/Smad inhibitors on the osteogenic differentiation of HBMSCs. Cells were incubated with osteogenic differentiation medium in the presence of KR-12 (1000 μg mL^−1^) along with an inhibitor of TGF-β/Smad (LDN-193189 HCL). (A) Western blot analysis results for the levels of phosphorylated Smad1/5, overall Smad1, Sma5 and Smad4 at 7 days. (B) The quantitative assay of P-Smad1/5/GAPDH (****P < 0.0001). (C) Entire plate views and micrographs of alkaline phosphatase (ALP) staining at 7 days and alizarin red staining at 21 days. (D) Quantitative evaluation of ALP activity and alizarin red staining results (****P < 0.0001). (E) BMP2, Runx2, ALP, COL-1, BSP, OSX, OCN and OPN mRNAs were subjected to quantitative real-time PCR analysis at 7 days. The expression levels were normalized to that of GAPDH (*P < 0.05, ***P < 0.001, ****P < 0.0001).

To further clarify the role of BMP/SMAD signaling in osteoblast differentiation, we blocked the BMP/SMAD signaling pathway using a TGF-β/SMAD inhibitor (LDN-193189 HCL). Western blot analysis was performed to observe SMAD phosphorylation levels after 7 days (Fig. 4A). Compared with the control group, treatment with the inhibitor significantly reduced the phosphorylation level, regardless of whether 1000 μg mL^−1^ KR-12 was added. The quantified western blot results are shown in Fig. 4B. As shown in Fig. 4C, blocking BMP/SMAD signaling decreased ALP and mineralization staining in the HBMSCs. Quantitative assays showed similar results (Fig. 4D). These results confirm that BMP/SMAD signaling plays a positive role in HBMSC osteogenesis. This observation was further supported by RT-PCR results (Fig. 4E), which showed that blocking the activation of BMP/SMAD signaling suppressed the expression of BMP2 and several osteoblast marker genes (RUNX2, ALP, COL-1, BSP, OCN, OPN, and OSX) at day 7.

### 5. BMP2 knockdown blocks the KR-12-induced osteogenic differentiation of HBMSCs

**Fig. 5:**
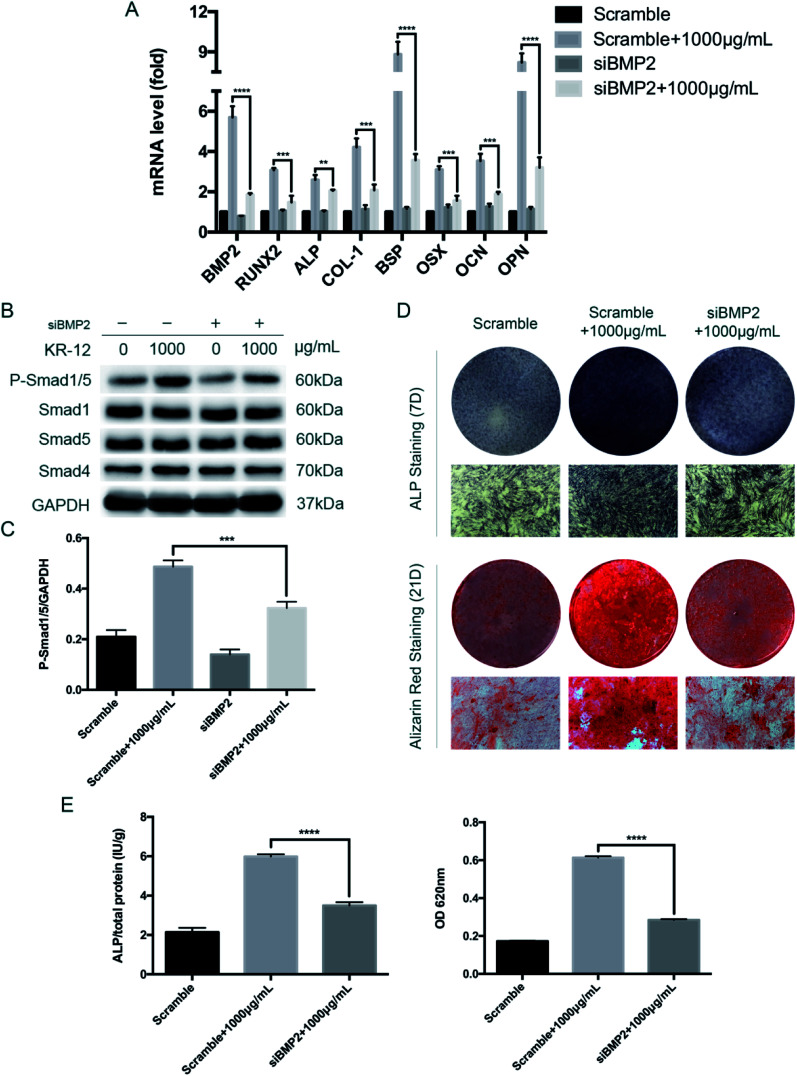
The effect of BMP2 knockdown on osteogenic differentiation of HBMSCs induced by KR-12 (1000 μg mL^−1^). (A) BMP2, Runx2, ALP, COL-1, BSP, OSX, OCN and OPN mRNAs were analyzed by quantitative real-time PCR at 7 days. The expression levels were normalized to that of GAPDH (**P < 0.01, ***P < 0.001, ****P < 0.0001). (B) Western blot analysis results for the levels of phosphorylated Smad1/5, overall Smad1, Sma5 and Smad4 at 7 days. (C) The quantitative assay of P-Smad1/5/GAPDH (***P < 0.001). (D) Entire plate views and micrographs of alkaline phosphatase (ALP) staining at 7 days and alizarin red staining at 21 days. (E) Quantitative evaluation of ALP activity and alizarin red staining results (****P < 0.0001).

To further evaluate the role of BMP2 in the KR-12-induced osteogenic differentiation of HBMSCs, we evaluated the effects of siRNA knockdown. As shown in Fig. 5A, BMP2 silencing at day 7 markedly decreased the expression of BMP2, RUNX2, ALP, COL1A1, OCN, OPN, and OSX, after significant elevation of these levels by 1000 μg mL^−1^ KR-12. After BMP2 knockdown, SMAD1/5 phosphorylation was also significantly decreased at day 7 (Fig. 5B and C). Further staining and quantitative assays of ALP (day 7) and Alizarin Red (day 21) was showed in Fig. 5D and E. Taken together, these results suggest that BMP2 mediates the KR-12-induced osteogenic differentiation of HBMSCs.

## Discussion

Bone infection and osteolysis caused by osteomyelitis are common clinical manifestations in orthopedics, and require the use of systematic and topical antibiotics.^21–23,34^ With the increased emergence of drug-resistant bacteria in clinical bone infections, traditional antibiotics are becoming increasingly inefficient in their treatment, and AMPs, with lower drug resistance probability and good antibacterial properties, are being increasingly investigated.^24^ AMPs are components of the innate immune system, and are considered potential alternatives to conventional antibiotics.^1,2^ AMP LL-37 is an important first line of defense against local infection and systemic pathogen invasion, and does not cause bacterial resistance.^6,7,12,13^ However, because of its long amino acid sequence, LL-37 is difficult to develop as a therapeutic agent against infectious diseases.^14^ As the minimal active fragment of LL-37, KR-12 has the advantages of low synthesis cost and low cytotoxicity, and could play a key role in the treatment of infections caused by drug-resistant bacteria.^14,20^

While antibiotics are commonly used to control infection in clinic, they do not promote new bone formation, and may even inhibit local osteogenesis, making them ineffective against another common clinical problem, infection-related osteolysis. Several studies have demonstrated that antibiotics, including gentamicin and vancomycin, inhibit osteoblast proliferation and osteogenic differentiation.^25,55^ Conversely, some small molecule peptides can promote osseointegration while controlling infection.^27^ Therefore, using AMPs to promote local osteogenesis could result in better treatment of osteomyelitis and consequent osteolysis. Our previous studies have shown that covalent bonding of KR-12 to the surface of titanium can decrease infection and promote the osteogenic differentiation of HBMSCs.^49^ BMSCs have been widely used for osteogenic differentiation research, since osteoblasts originate from BMSCs.^56^ Thus, human primary BMSCs were chosen in the present study. To understand whether KR-12 alone affects osteogenic differentiation, here we have studied its effects without titanium.

The potential cytotoxic effects of KR-12 were investigated before examining its effects on osteogenic differentiation. Our investigation verified that KR-12 was non-cytotoxic to HBMSCs at concentrations up to 1000 μg mL^−1^, which was therefore the maximum concentration used in subsequent osteogenic differentiation experiments. Cell cycle analysis demonstrated that KR-12 did not affect cell cycle progression, meaning that KR-12 did not significantly influence proliferation at the concentrations tested. KR-12 also had no effect on apoptosis. These results demonstrate that KR-12 has no adverse effects on the growth and biological functions of HBMSCs, providing the basis for its subsequent application.

Our results demonstrate that KR-12 can enhance the osteogenic and mineralization potential of HBMSCs in a dose-dependent manner. When the concentration of KR-12 reached 1000 μg mL^−1^, the ability to promote osteogenic differentiation significantly increased, meaning that KR-12 may most efficiently promote osteogenic differentiation at high concentrations. To precisely detect osteogenic marker expression after KR-12 stimulation, measurements were performed at different stages of osteogenic differentiation. In the early stages (the first week of osteogenic differentiation), ALP, RUNX2, COL-1, and BSP were increased. In contrast, late-stage markers, including OSX, OPN, and OCN were increased in the second week.^57^ RT-PCR results at different stages indicated that KR-12 enhanced osteogenic gene transcription. This effect was also more pronounced at high concentrations of KR-12. Taken together, these results indicate that KR-12 stimulates the osteogenic differentiation of HBMSCs *in vitro*.

Several pathways are reported to be involved in osteogenic differentiation, including the Wnt/b-catenin,^58^ MAPK,^59^ PI3K^60^ and estrogen receptor pathways.^61^ As BMP2 expression increased with KR-12 stimulation (Fig. 3A), we hypothesized that the stimulatory effects of KR-12 on osteoblastic differentiation may be exerted through activation of the BMP/SMAD signaling pathway. BMPs, which belong to the TGF-β superfamily, have potent osteogenic effects and control osteoblast differentiation during osteogenesis. BMP2 promotes differentiation by enhancing intracellular ALP activity, as well as OCN and COL1A1 protein synthesis.^62^ The effects of BMPs are mediated by receptor serine/threonine kinases and the downstream transcription factors SMAD1/5/9. Upon phosphorylation by the receptors, SMAD1/5/9 form a complex with SMAD4, translocate to the nucleus, and regulate the transcription of target genes associated with differentiation.^42,63^ Several natural or chemical agents, including rapamycin, daidzein, and syringetin, have been reported to induce osteoblastic differentiation by inducing BMP and/or SMAD signaling.^64–66^ This study showed that BMP2 expression was enhanced and SMAD1/5 phosphorylation as significantly increased in KR-12-treated HBMSCs. KR-12-mediated SMAD1/5 activation was blocked by the TGFβ/SMAD antagonist LDN-193189 HCL, and HBMSC differentiation was also attenuated. Furthermore, BMP2 siRNA decreased SMAD1/5 phosphorylation and inhibited KR-12-enhanced osteogenic differentiation. Hence, BMP2/SMAD signaling plays an important role in KR-12-mediated HBMSC differentiation.

## Conclusion

In conclusion, KR-12 promoted the osteogenic differentiation of HBMSCs in a dose-dependent manner, significantly increasing osteogenesis at 1000 μg mL^−1^. Furthermore, the BMP/SMAD signaling pathway might play a crucial role in the induction of osteogenic differentiation by KR-12. The present study has some limitations. First, although primary HBMSCs were used, the results obtained in this study are unlikely to be fully representative of the *in vivo* effects. Therefore, the translational relevance of this study should be confirmed in more bone formation-related cell models, like osteoclasts, as well as *in vivo*. Second, activation of BMP/SMAD signaling may not be the only mechanism through which KR-12 enhances osteogenesis. More studies are needed to explore the effects of KR-12 on other potential signaling pathways. In addition, given the potential for AMPs in the treatment of osteomyelitis and other infections, the discovery of additional peptides with good antibacterial properties and the ability to modulate osteogenesis would be beneficial. Taken together, the results in this study suggest that KR-12 may be a useful agent for the prevention and treatment of local osteomyelitis and related osteolysis.

## Authors' contributions

Hui Li, Shutao Zhang, Bin'en Nie, Zhe Du and Teng Long carried out the experiments. Hui Li, wrote the manuscript. Bing Yue and Teng Long designed the experiments. Bing Yue revised the manuscript. All authors reviewed the manuscript.

## Conflicts of interest

The authors declare no competing interests linked to this study.

## Supplementary Material
